# Assessment of human health risks posed by toxic heavy metals in Tilapia fish (*Oreochromis mossambicus*) from the Cauvery River, India

**DOI:** 10.3389/fpubh.2024.1402421

**Published:** 2024-11-13

**Authors:** Nikita Gupta, Sathiavelu Arunachalam

**Affiliations:** ^1^School of BioSciences and Technology, Vellore Institute of Technology, Vellore, India; ^2^VIT School of Agricultural Innovation and Advanced Learning, Vellore Institute of Technology, Vellore, India

**Keywords:** aquatic pollution, Cauvery River, environmental pollution, heavy metal, health risk assessment, Tilapia fish, toxicity

## Abstract

Heavy metal toxicity is a serious threat to human health due to its bioaccumulation, biomagnification, and persistent nature in the environment including aquatic systems. In the recent past, heavy metal contamination in the environment has occurred due to various anthropogenic sources. The concentration of potentially toxic heavy metals was determined by Atomic Absorption Spectroscopy in Tilapia (*Oreochromis mossambicus*), a highly farmed and consumed fish species in southern parts of India. The mean levels of Fe were found to be higher in major organs of the fish with the highest levels in liver (Mean 1554.4 ± 1708.7 mg/kg) and lowest in the muscles (Mean 130.757 ± 33.3 mg/kg). Correlation Matrix analysis revealed relationships between the occurrence of various heavy metals in different organs of fish and indicated similar origins and chemical properties. Target hazard quotient for Cd, Co, Pb, and Cr in the Liver, Co and Cr in the Gills, and Co in Muscle were > 1 for adults, which showed a significant health risk from the combined effects of these metals. The potential health risk to humans, according to the cancer risk (CR) assessment is attributed mainly to Cd and Cr levels. Overall, moderate fish consumption is advised to limit the bioaccumulation of heavy metals over prolonged exposure and associated health risks.

## 1 Introduction

The anthropogenic pollution of freshwater bodies is of major concern globally and so is in India (1). In this study, the authors emphasized the impact of anthropogenic activities on the fish fauna in the Ujjani Reservoir in Maharashtra, India. They also reported higher levels of heavy metals in the fish from the reservoir than normal. India is a country with rich biodiversity along with a number of freshwater reserves in the form of rivers, lakes, ponds, etc. However, in the past decade, there has been an indiscriminate discharge of industrial and agricultural pollutants into the water bodies through various anthropogenic activities creating severe deterioration of water quality, thereby affecting aquatic life (2–5). Over the years, efforts have been made by different environmental protection agencies to control the amount of pollutants dumped into the rivers. The government also supported some studies on anthropogenic activities and their influence on heavy metals in Indian rivers (6). Nevertheless, much needs to be done to restore the water bodies to their native state and mitigate the impact of pollution on aquatic and human health.

Trace heavy metals present in the aquatic ecosystem are released through agriculture and industries which accumulate at various trophic levels of the food chain. However, this accumulation can slowly reach hazardous levels and turn into an environmental problem. There have been several studies about the prominent presence of heavy metals on sediments and their impact on seawater, and aquatic organisms (7–9). A few studies on rivers in India have also discussed the potential impacts of heavy metals on humans (2, 4). Several studies have explored the bioaccumulation of heavy metals in fishes (10–12). The consensus seems to be that fishes in heavy metal-contaminated areas tend to absorb certain heavy metals in ionic forms from their immediate environment. Environmental factors such as pH and temperature modulate this uptake. The gills and skin, directly exposed to the contaminated water act as hotspots for its absorption. Following the uptake, heavy metals are transported to various organs via blood flow where the coupling of heavy metals with various proteins takes place. Although, fishes do regulate their body metal concentration to some degree via excretion through gills, skin, kidneys, and bile. Studies all around the world have reported various risks and health hazards associated with fishes’ bodies which results from disturbances in normal cellular activities, oxidative damage to biological macromolecules such as DNA and RNA caused by heavy metals (12, 13). Accumulation of heavy metal also depends on the habitat of the fishes, sedimentary fishes that stay in stagnant water in muddy streams that are contaminated have been reported to have higher heavy metal content (14). Heavy metal accumulation has a multidirectional toxic effect on fish. In some cases, it manifests changes in the physiochemical processes of the body. Structural lesions and functional disturbances could also result from the bioaccumulation of metals (15).

Eating fish contaminated with heavy metals can have significant adverse effects on human health. Heavy metals such as cadmium, mercury, lead, and arsenic, when accumulated in fish tissues, can pose severe health risks when these fish are consumed by humans. These metals are known to be potent carcinogens and mutagens. Cadmium is known to be primarily toxic to the kidneys, cadmium can accumulate in the human body over time, potentially leading to kidney damage. Mercury is shown to affect the central nervous system, and high exposure can lead to neurological and behavioral disorders. Mercury is particularly dangerous to pregnant women as it can affect fetal development. Lead exposure can cause damage to the nervous system, kidney function, and the cardiovascular system. In children, lead exposure can result in developmental issues and reduced cognitive function. Arsenic exposure can lead to skin lesions, cancer, cardiovascular diseases, and diabetes. Long-term exposure to heavy metals through contaminated fish consumption can result in chronic conditions such as Alzheimer’s disease, Parkinson’s disease, muscular dystrophy, multiple sclerosis, and other neurological and muscular diseases. Allergies and increased cancer risk are also associated with prolonged heavy metal exposure (16, 17).

In India, fishes are considered a staple food source and the *per capita* consumption in some states reaches as high as 29.29 kg/year (18). Most of the fishing needs are met with inland fish production which is more susceptible to various sources of water pollution. Inland fishes thus are more hazardous to human health. Consumption of a heavy metal-contaminated diet can lead to the depletion of vital nutrients which can cause severe damage to immunological defenses, malnutrition-related disabilities, and impaired psychosocial behavior. Hence, the regular risk assessment of these heavy metals intake via diet is of utmost concern (19–21).

Mozambique Tilapia (*Oreochromis mossambicus*) is a variety of edible freshwater fish that has an omnivorous nature with high local demand in several developing countries like Malaysia. Commercial production of Tilapia fish takes place in almost 10 countries around the world (22, 23). It is one of the most important farmed fishes in the world next to carp and salmon. The low cost and high production coupled with its suitability for aquaculture and marketability make it a lucrative option for people in developing countries. Tilapias can adapt to various environmental conditions and demonstrate higher resistance to diseases but are susceptible to leachate toxicity (24). The wide acceptability of the Tilapias is evident from the production boost over the last decade resulting in a four-fold increase in its production (25, 26).

The present study is focused on monitoring the levels of various heavy metals in Tilapia, an exotic fish of the Cauvery River. Tilapia fish (*Oreochromis mossambicus*) is widely popular and highly consumed in southern parts of India (27, 28–33). It has high nutritional value and is a rich source of proteins, amino acids, vitamins, minerals, PUFA (polyunsaturated fatty acids), and some essential heavy metals. Heavy metals such as Manganese, Zinc, and Iron in optimal concentrations are supportive for the normal growth of humans and animals (34). However, some heavy metals such as arsenic, cadmium, mercury, lead, etc. do not play any beneficial role in the biological systems and can lead to a variety of diseases (1). Despite fish aiding in fulfilling our food, particularly protein demand, which in turn reduces the burden on agriculture, the presence of high amounts of essential as well as harmful heavy metals poses a serious risk to human health.

In the current scenario, Genetically Improved Farmed Tilapia (GIFT), is considered a candidate species for aquaculture in India. Its affordability and animal protein content make it a fish of choice among consumers (35). Various business organizations positively argue for the expansion of tilapia production in India to meet fish and marine export goals. This species has a relatively high survival rate and faster growth makes it lucrative for small-scale and large-scale GIFT farmers (36, 37). On the other hand, environmentalists argue over responsible aquaculture and strict regulations. Overall, Tilapia is currently seen as the next billion-dollar enterprise in India. Our study aims to understand and estimate the heavy metal content in this widely consumed, and important fish species in India, whose consumption is further likely to be increased in the coming years (34). Therefore, a study on this species not only provides us with the overall scenario of heavy metal load in the Cauvery River but also acts as a reference for further studies in the upcoming years with the aim to determine the levels of heavy metal concentrations in various organs of Tilapia fish.

## 2 Materials and methods

### 2.1 Ethics statement

Our study did not require ethical board approval because it did not contain human or animal trials. The fish used in this study were procured dead from local fishermen. All surgical operations were performed on dead fish. Care was taken to ensure that the least number of fish was utilized to reach satisfactory statistical conclusions.

### 2.2 Sample collection

**Figure 1:**
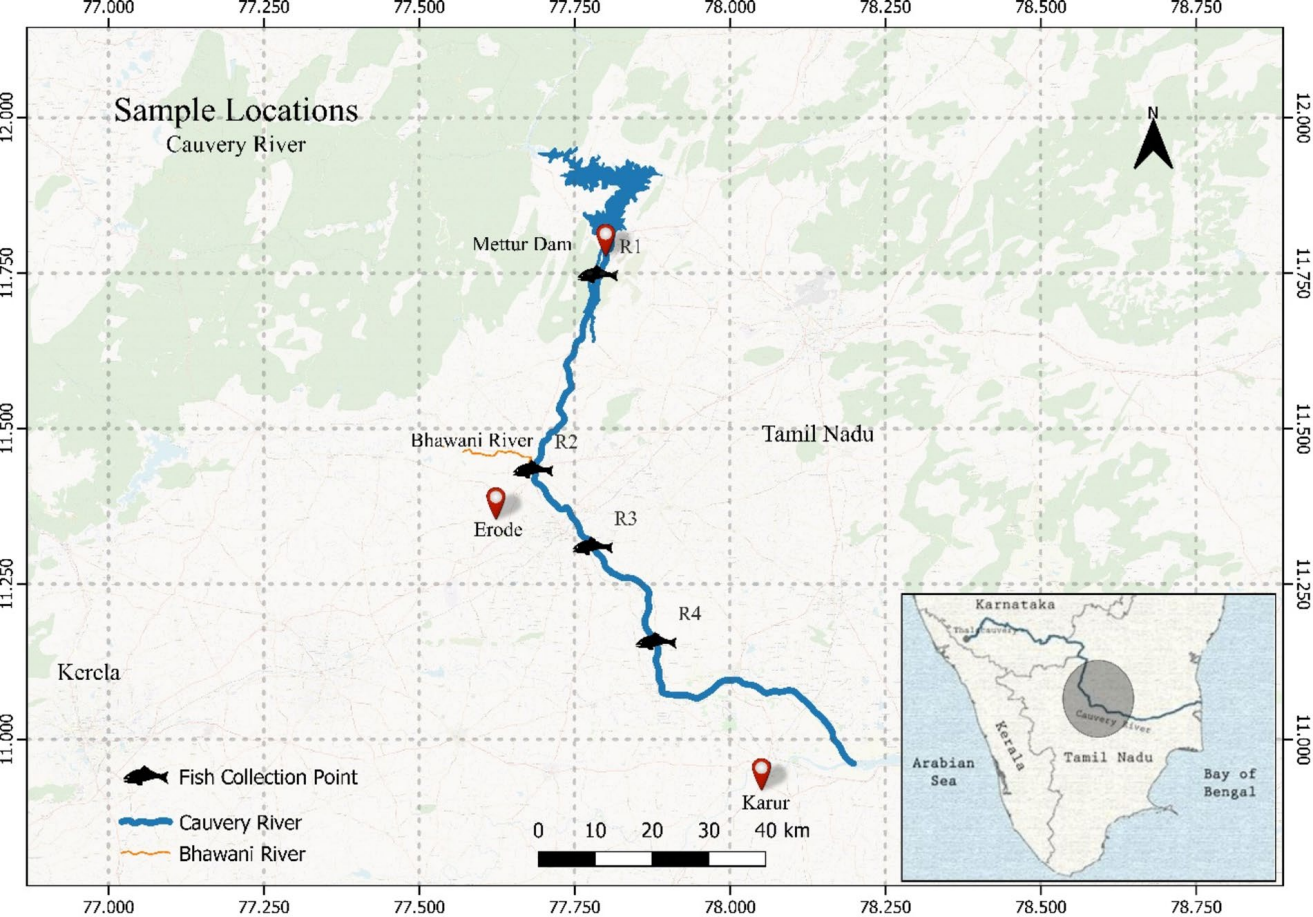
Location map of the study area showing sampling locations and important places.

The Cauvery River in southern India is vital for agriculture, industry, and urban populations in Karnataka and Tamil Nadu, but it faces significant metal contamination due to anthropogenic activities. Agricultural runoff, industrial discharges, and urban waste contribute to the presence of heavy metals like lead, chromium, and cadmium in the river (38). These metals bio-accumulate in fish, posing health risks to humans and disrupting aquatic ecosystems (39). Effective mitigation requires stringent regulatory measures, sustainable agricultural practices, and robust waste management systems, alongside regular monitoring and community engagement (40). Figure 1 represents the four sampling sites near the Erode region across the Cauvery River in Tamil Nadu state in India. The sampling sites were carefully chosen to cover the maximum stretch of the river possible, there is also quite a few textile industries and other industries in the area near Erode. The sampling stations were as follows: R1 (11°74′75.34” N; 77°78′69.66″ E); R2 (11°43′36.68” N;77°68′27.9″ E); R3 (11°31′02.94” N;77°77′87.36″ E); R4 (11°15′72.31” N;77°88′11.61″ E). A total of sixteen (16) fresh and adult, Tilapia fish involved in the study were purchased from the local fishermen in each area depending on the sites from where the fish were planned to be sampled (41, 42). Samples collected were the maximum feasible given only one species of fish was targeted with similar size and body weight. All the fish were dead and stored in ice after purchase and carried forward for further analysis. On arrival, all the samples were labeled and stored at -20°C for further analysis. All 16 samples collected were analyzed as per the standard protocols published and raw data is provided as Supplementary file S1.

### 2.3 Sample analysis

The fishes collected had average lengths and weights of 17.7 cm (measured with a ruler) and 112.5 g (measured with a weighing balance (Mettler Toledo ME204)), respectively Supplementary file S1. Fish sample collection was done in the pre-monsoon period. A stainless-steel scalpel was used to dissect various portions of the raw sampled fish, which were Muscle, Liver and Gills and taken for further analysis. A digestion tube containing 0.1–1 g of the sample (dry weight) was weighed, and 5 mL of HNO_3_ and 5 mL of H2SO4 were then added. The reaction was allowed to complete, and when it did, the tubes were put in a hot-block digestion device from BioBee® with 12 slots and temperature control and heated for 30 min at 60°C before being heated again to 150°C. When the samples’ color turned black, the tubes were taken out of the experiment. After allowing the tubes to cool, 1 mL of H_2_O_2_ was added. The tubes were repositioned on the block after a strong reaction. Slowly adding H2O2 made the sample’s solution appear clear. The tubes were taken out, and the sample solution was diluted with deionized water to a volume of 50 mL (12, 43).

The heavy metals (Cr, Cd, Fe, Ni, Zn, Co, Pb, and Cu) concentration was determined using a Varian - Atomic Absorption Spectrophotometer (AA240 Atomic Absorption Spectrometer). To test the instrument’s accuracy, standard solutions, and samples were run simultaneously. Analytical conditions for the measurement of the heavy metals in the sample using AAS were tabulated in Table 1. All the fish samples were measured in triplicates and the mean was taken forward for further calculation and reported as it is, as no randomization was performed given the samples were collected directly from rivers. All chemicals and reagents were of analytical reagent-grade quality. Before use, all glass and plastic ware were soaked in 14% HNO_3_ for 24 h. The washing was done with distilled water. Measurements were done simultaneously for each group to avoid batch effects if any. Data analysis was performed on a Spreadsheet and GraphPad Prism (Version 8.0).

**Table 1:** Analytical conditions for the measurement of the heavy metals in the sample using AAS.

Heavy Metal	Wavelength	Slit (nm)	Lamp current (mA)
Cd	228.8	0.5	4
Cu	222.6	0.2	4
Cr	428.9	0.5	7
Fe	372.0	0.2	5
Pb	283.3	0.5	5
Zn	213.9	1	5
Co	304.4	0.5	7
Ni	341.5	0.2	4

### 2.4 Relevant parameter estimations

#### 2.4.1 Calculation of heavy metal (HM) in tissues

The concentration of minerals is calculated according to the equation given below (44),


$$\begin{array}{l} \text{Mineral}\left(\frac{\text{mg}}{L}\right)=\\ \text{Reading}\;\text{of}\;\text{mineral}\;\text{in}\;AAS\times \frac{50\left(\text{made}-\text{up}\;\text{volume}\right)}{\text{weight}\;\text{of}\;\text{sample}\left(g\right)} \end{array}$$

#### 2.4.2 Pollution index (PI)

To determine the PI of the elements, statistical analysis was performed on the elemental concentrations in fish samples. The PI is the ratio of element x concentration in the sample to the element’s maximum allowable level (41).


$$\text{PI}\left(x\right)=\frac{\text{Metal}\;\text{concentration}\;\text{in}\;\text{the}\;\text{sample}}{\text{Permissible}\;\text{limit}\;\text{or}\;\text{background}\;\text{value}}$$

It is generally accepted that if an element’s PI value is more than 1.0, the element is highly likely to have contaminated the sample and may even be dangerous at the amount it is present.

#### 2.4.3 Estimated daily intake (EDI)

The estimated daily intake (EDI) was calculated using the following formula (45).


$$EDI=\frac{E_{F}\times E_{D}\times \text{FIR}\times C_{F}\times C}{W_{\text{AB}}\times T_{A}}\times 10^{-3}$$

where ED, EF, *CF*, WAB, FIR, C, and TA stand for the exposure duration (60 years), exposure frequency (365 days annually), conversion factor (0.208) to convert fish’s dry weight to wet weight, average adult weight of the body (70 kg), consumption rate (25.2 g per day), heavy metal concentrations in fish’s muscle tissues, and average exposure time, respectively (45–49). The daily intake values were compared with reference values established by the United States Environmental Protection Agency (50), making the USEPA the legislation that will serve as the bibliographic tool in the comparative analysis. All the calculations in this study were made for adult human with standard fish intake over lifetime.

#### 2.4.4 Target hazard quotient (THQ) or non-carcinogenic health hazard

THQ measures the risk of side events other than cancer by comparing the exposure dosage to the reference dose (RfD). The exposure level is lower than the RfD if it is less than 1. This suggests that lifetime unfavorable effects are unlikely to result from daily exposure at this level and vice versa. Standard assumptions from the integrated USEPA risk study were used to construct the dosage estimations (41, 50).

The target hazard quotient (THQ) was estimated using the following formula.


$$THQ=\frac{EDI}{RfD}$$

In this study, the total THQ was calculated as the arithmetic sum of the individual THQ values of the metal of concern (51).


$$\begin{array}{l} \text{Total}\;THQ\left(\text{TTHQ}\right)=\\ THQ\;\left(\text{toxicant}\;1\right)+THQ\;\left(\text{toxicant}\;2\right)+\ldots THQ\;\left(\text{toxicant}\;n\right) \end{array}$$

#### 2.4.5 Carcinogenic risk or cancer risks (CR)

The Cancer Risk over a lifetime of Cd, Pb, and Cr exposure was calculated by applying the following formula (45, 46).


CR=EDI×CSF

### 2.5 Data analysis

#### 2.5.1 Principal component analysis (PCA)

Principal Component Analysis was used to reduce the dimensionality of the dataset. It was used to identify patterns in the distribution of heavy metals across different fish organs. The analysis was performed in GraphPad Prism version V.10. Principal Component Analysis transformed the original variables into a new set of uncorrelated variables which are also known as principal components these components are ordered by the amount of variance they explain in the data. This method allows for the visualization of the data structure and the identification of the most significant variables contributing to the observed variance. PCA can identify linear relationships between different inter-associated variables. PCA extracts eigenvalues and eigenvectors from the covariance matrix of the original associated variables. The principal component (PC) is an orthogonal variable, which is attained by multiplying the eigenvector with the original associated variables. The first few principal components, which capture the majority of the variance, were used to interpret the relationships between the heavy metal concentrations and the fish organs. Since there were various factors influencing the accumulation of heavy metals in fish muscles, Principal Component Analysis was used to explore the effects of size and body weight of fish on the accumulation of heavy metals in different organs using the analyzed heavy metal concentrations matrix.

#### 2.5.2 Correlation matrix analysis

A correlation matrix was computed to analyze the associations between the various concentrations of heavy metals within various organs of fish. The correlation matrix was also analyzed with GraphPad Prism v.10. The correlation matrix is a summary of all the pair-wise correlations between the variables, measured by means of Pearson correlation coefficients. Correlations are meaningful, and a heatmap is used to see significant correlations in the matrix. A correlation coefficient-the value ranging from −1, for a perfect inverse relation, through 0, for no relation, to 1, for a perfect direct relation-evaluates any two variables on a scale from −1 to 0 to 1. High values and positive significant correlation may indicate chemical affinity between the metals, common genetic origin and /or a background level present in the samples; negative correlation might point toward different origins for the metals or a non-chemical relationship. Considering the various trends in the level of correlation, we explained the strength of the correlation of heavy metals within each organ separately. Beyond the statistical tools used in this study, additional hidden features and strength of the data set could be unearthed by the linkage of variables using non-linear tools.

## 3 Results

### 3.1 Heavy metal concentrations

**Figure 2:**
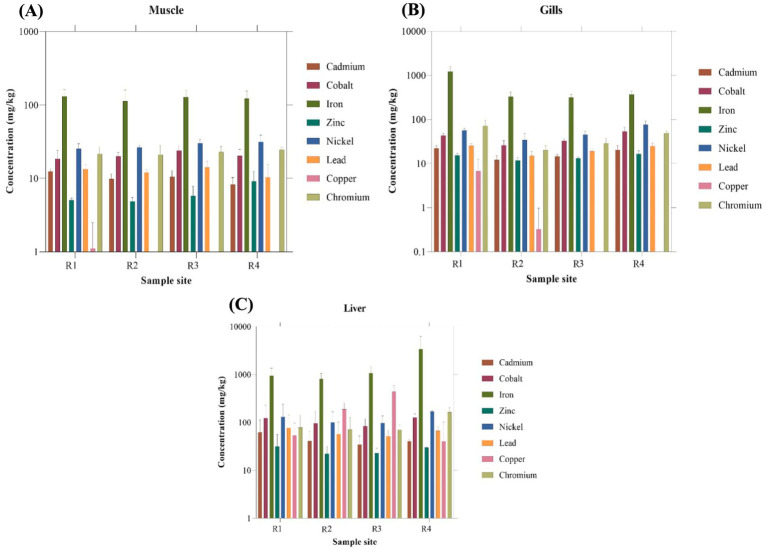
Concentration (mg/kg) of heavy metals in **(A)** Muscle, **(B)** Gills, and **(C)** Liver of *Oreochromis mossambicus.*

The metal concentrations in various body organs of Tilapia fish species are presented in Figure 2. Since most of the heavy metal concentrations did not significantly differ between the sample sites (R1 – R4), all four sites were combined for further analyses and correlation studies. In fish samples, iron concentration was found to be relatively higher than the other metals. The heavy metal concentration varied across different organs in the following sequence: Muscle Fe > Ni > Cr > Co > Pb > Cd > Zn > Cu, Gills Fe > Ni > Co > Cr > Pb > Cd > Zn > Cu, Liver Fe > Ni > Cr > Co > Cu > Pb > Cd > Zn. The maximum Fe levels were detected in the Liver (Mean: 1554.4 ± 1708.7 mg/kg) of Tilapia, while the minimum Fe levels were observed in the muscles (Mean: 130.757 ± 33.3 mg/kg). Muscles contained a low Fe level compared to the other organs. Apart from Iron other heavy metals like Cr, Co, Pb, and Cd were also found to be well above the standard permissible limits (Table 2) for the respective heavy metals in respective tissue samples.

**Table 2:** The permissible limit of heavy metals in fisheries.

Institute/Organization	Zn	Cu	Pb	Ni	Cd	Mn	Fe	Cr	Reference
MFA (Malaysian Food Act)	100	30	2	-	1	–	–		MFA (65)
FAO (Food and Agriculture Organization) (1983)	30/40	30	0.5	-	0.5	–	–		FAO (66),
EC (Commission of the European Communities)	–	–	0.2–0.4	-	0.05	–	–	–	EC (67)
USFDA (Food and Drug Administration)	–	–	0.5	-	0.01–0.21	–	–		USFDA (68)
WHO (1989)/(2013)	100/5	30/2.25	2	–	1	1/0.5	100/0.30	–	Mokhtar et al. (69)
England	50	20	2	–	0.2	–	–		Contaminants (70)
FAO/WHO limits	40	30	2	–	0.5	–			Joint and Additives (71)
Median International Standard (Tolerable levels) (ug/g)	45	20	2	–	0.3	–	–	1	Phillips (72), Senarathne (73), and Senarathne and Pathiratne (74)
USEPA (United State Environmental Protection Agency) (ug/g)	5	2.25	0.11	–	0.01	0.02	0.5	–	Anim-Gyampo et al. (75)
WPCL (Water Pollution Control Legislation)	4.25	2	0.05	–	0.03	0.02	0.45	–	Anim-Gyampo et al. (75)

### 3.2 Pollution index (PI) of the heavy metals

To assess the degree of contamination or pollution linked to the obtained fish samples, the PI of heavy metals in the Tilapia fish samples was determined. Table 3 displays the PI values for the analyzed metals. We found that Zn and Cu pollution index were lower than 1 across the various organs. Fe, Cd, Pb, Cr, Ni, and Co had high pollution index across all the organs. Overall, the pollution index values for heavy metals in Gills and Liver far surpassed those in Muscle.

**Table 3:** Pollution index (PI) of the studied heavy metals in the Tilapia Fish samples.

	Pollution index muscle				Pollution index liver				Pollution index gills			
HM	R1	R2	R3	R4	R1	R2	R3	R4	R1	R2	R3	R4
Cd	12.42	9.83	10.47	8.22	62.05	41.02	34.35	40.63	22.05	12.00	14.42	20.20
Co	65.82	71.52	85.77	72.27	440.29	342.92	296.75	450.00	154.15	92.05	117.59	190.85
Fe	261.51	225.93	255.35	243.59	1878.87	1615.56	2151.31	6789.50	2430.51	658.47	631.99	737.07
Zn	0.05	0.05	0.06	0.09	0.32	0.22	0.23	0.30	0.15	0.12	0.13	0.17
Ni	50.64	52.75	60.05	62.74	260.12	197.54	192.62	334.25	113.16	68.92	91.55	153.61
Pb	6.72	6.00	7.05	5.19	38.64	28.63	25.82	33.44	12.69	7.44	9.49	12.38
Cu	0.04	0.00	0.00	0.00	1.79	6.30	14.70	1.35	0.23	0.01	0.00	0.00
Cr	21.40	21.01	22.92	24.44	77.82	70.62	69.79	162.69	72.00	20.27	28.69	48.57

### 3.3 Human health risk assessment

#### 3.3.1 Estimation of estimated daily intake (EDI) and target hazard quotient (THQ)

According to the United States Environmental Protection Agency’s (50), recommended oral reference dose is shown in Table 4. Our results showed that the EDI for the investigated metals was lower than the RfD (oral reference dose) with some exceptions which are Liver (Cd, Co, Pb, and Cr), Gills (Co and Cr), Muscle (Co). The THQ of each metal from ingestion of Tilapia was generally less than 1 except in the Liver (Cd, Co, Pb, and Cr), Gills (Co and Cr), and Muscle (Co) (see Table 5). THQ values for Cd, Co, Pb, and Cr in the Liver, Co and Cr in Gills, and, Co in Muscle were > 1 for adults (see Table 6).

**Table 4:** Reference dose (RfD) and cancer slope factor (CSF) for different metals reported in the literature.

Metal	RfD	CSF (mg/kg/day)	Reference
Cd	0.001	6.3	Mohammadi et al. (64) and Adebiyi et al. (41)
Co	0.0003		Saha et al. (49)
Fe	0.3		Adebiyi et al. (41)
Zn	0.3		Adebiyi et al. (41)
Ni	0.02		Miri et al. (45)
Pb	0.004	0.0085	Mohammadi et al. (64) and Adebiyi et al. (41)
Cu	0.04		Adebiyi et al. (41)
Cr	0.003	0.5	Mohammadi et al. (64) and Adebiyi et al. (41)

**Table 5:** Calculation of adult’s estimated daily intake (EDI) for identified elements from eating tilapia fish.

	EDI muscle				EDI gills				EDI liver			
HM	R1	R2	R3	R4	R1	R2	R3	R4	R1	R2	R3	R4
Cd	0.0009	0.0007	0.0008	0.0006	0.0017	0.0009	0.0011	0.0015	0.0046	0.0031	0.0026	0.0030
Co	0.0014	0.0015	0.0018	0.0015	0.0032	0.0019	0.0025	0.0040	0.0092	0.0072	0.0062	0.0094
Fe	0.0098	0.0085	0.0096	0.0091	0.0910	0.0247	0.0237	0.0276	0.0703	0.0605	0.0805	0.2542
Zn	0.0004	0.0004	0.0004	0.0007	0.0011	0.0009	0.0010	0.0012	0.0024	0.0017	0.0017	0.0022
Ni	0.0019	0.0020	0.0022	0.0023	0.0042	0.0026	0.0034	0.0058	0.0097	0.0074	0.0072	0.0125
Pb	0.0010	0.0009	0.0011	0.0008	0.0019	0.0011	0.0014	0.0019	0.0058	0.0043	0.0039	0.0050
Cu	0.0001	0.0000	0.0000	0.0000	0.0005	0.0000	0.0000	0.0000	0.0040	0.0141	0.0330	0.0030
Cr	0.0016	0.0016	0.0017	0.0018	0.0054	0.0015	0.0021	0.0036	0.0058	0.0053	0.0052	0.0122

**Table 6:** Calculation of target hazard quotient (THQ) for analyzed metals from Tilapia fish consumption by adults.

	THQ Muscle				THQ Gills				THQ Liver			
HM	R1	R2	R3	R4	R1	R2	R3	R4	R1	R2	R3	R4
Cd	0.930	0.736	0.784	0.616	1.651	0.898	1.080	1.513	4.646	3.072	2.572	3.042
Co	4.600	4.998	5.994	5.050	10.773	6.433	8.218	13.338	30.771	23.966	20.740	31.450
Fe	0.033	0.028	0.032	0.030	0.303	0.082	0.079	0.092	0.234	0.202	0.268	0.847
Zn	0.001	0.001	0.001	0.002	0.004	0.003	0.003	0.004	0.008	0.006	0.006	0.007
Ni	0.095	0.099	0.112	0.117	0.212	0.129	0.171	0.288	0.487	0.370	0.361	0.626
Pb	0.252	0.225	0.264	0.194	0.475	0.279	0.355	0.463	1.447	1.072	0.967	1.252
Cu	0.002	0.000	0.000	0.000	0.013	0.001	0.000	0.000	0.101	0.354	0.825	0.076
Cr	0.534	0.524	0.572	0.610	1.797	0.506	0.716	1.212	1.942	1.763	1.742	4.061
**TTHQ**	**6.446**	**6.611**	**7.760**	**6.621**	**15.228**	**8.331**	**10.623**	**16.910**	**39.636**	**30.803**	**27.481**	**41.361**

Total Target Hazard Quotient (TTHQ).

#### 3.3.2 Calculation of the cancer risk (CR) for cd, Pb, and Cr

**Figure 3:**
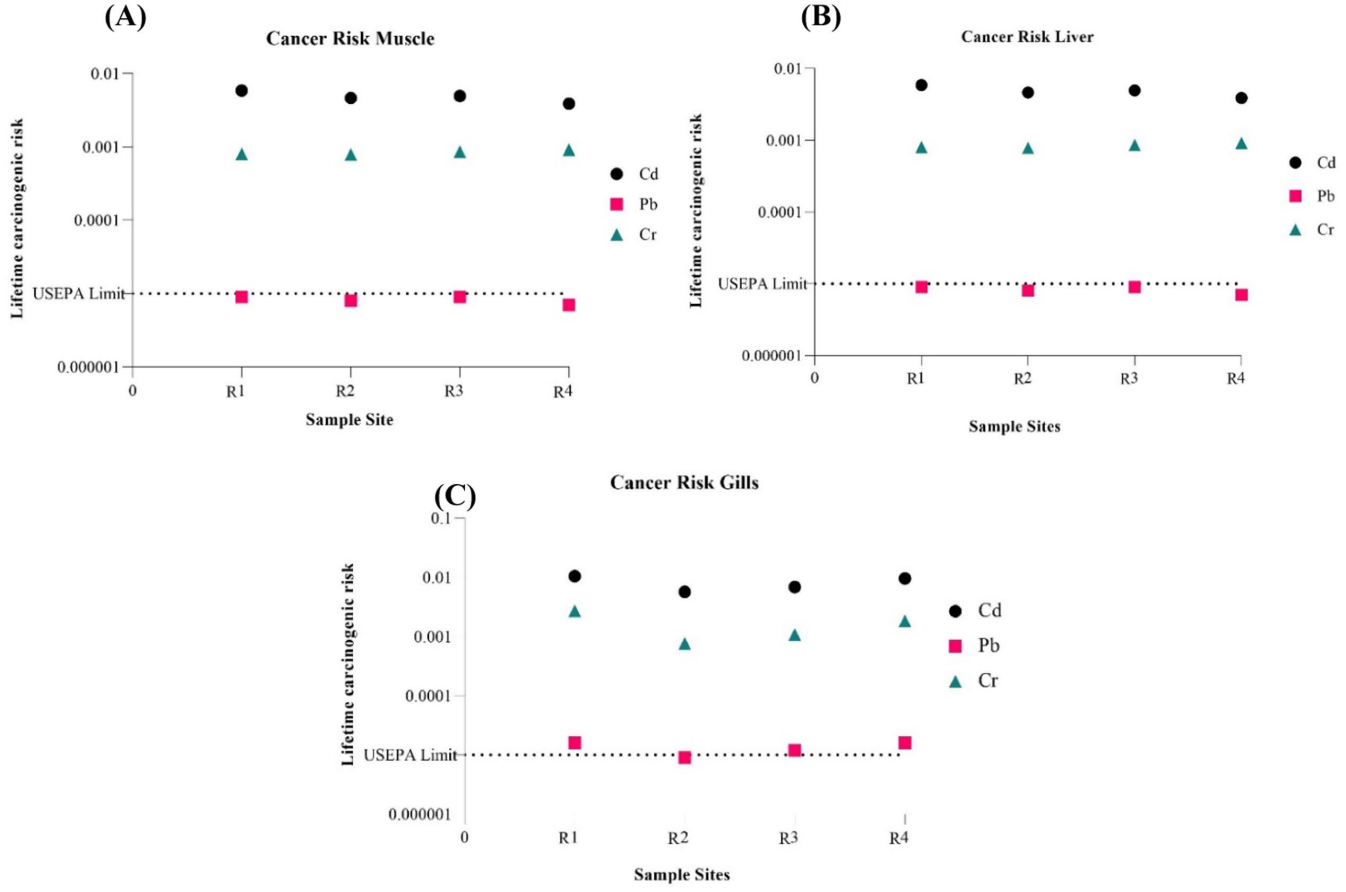
Estimated cancer risk for analyzed metals associated with consumption of **(A)** Muscle, **(B)** Liver, and **(C)** Gills from *Oreochromis mossambicus.*

Figure 3 showcases the estimated cancer risk factors for Cd, Pb, and Cr. The USEPA has assigned a 10–5 acceptable limit for the lifetime carcinogenic risk. Based on the findings, the muscle, liver, and gills Pb cancer risk factor determined in this study is within the established tolerable level. However, Cd and Cr are higher than the set tolerable limit.

### 3.4 Results of principal component analysis (PCA)

**Figure 4:**
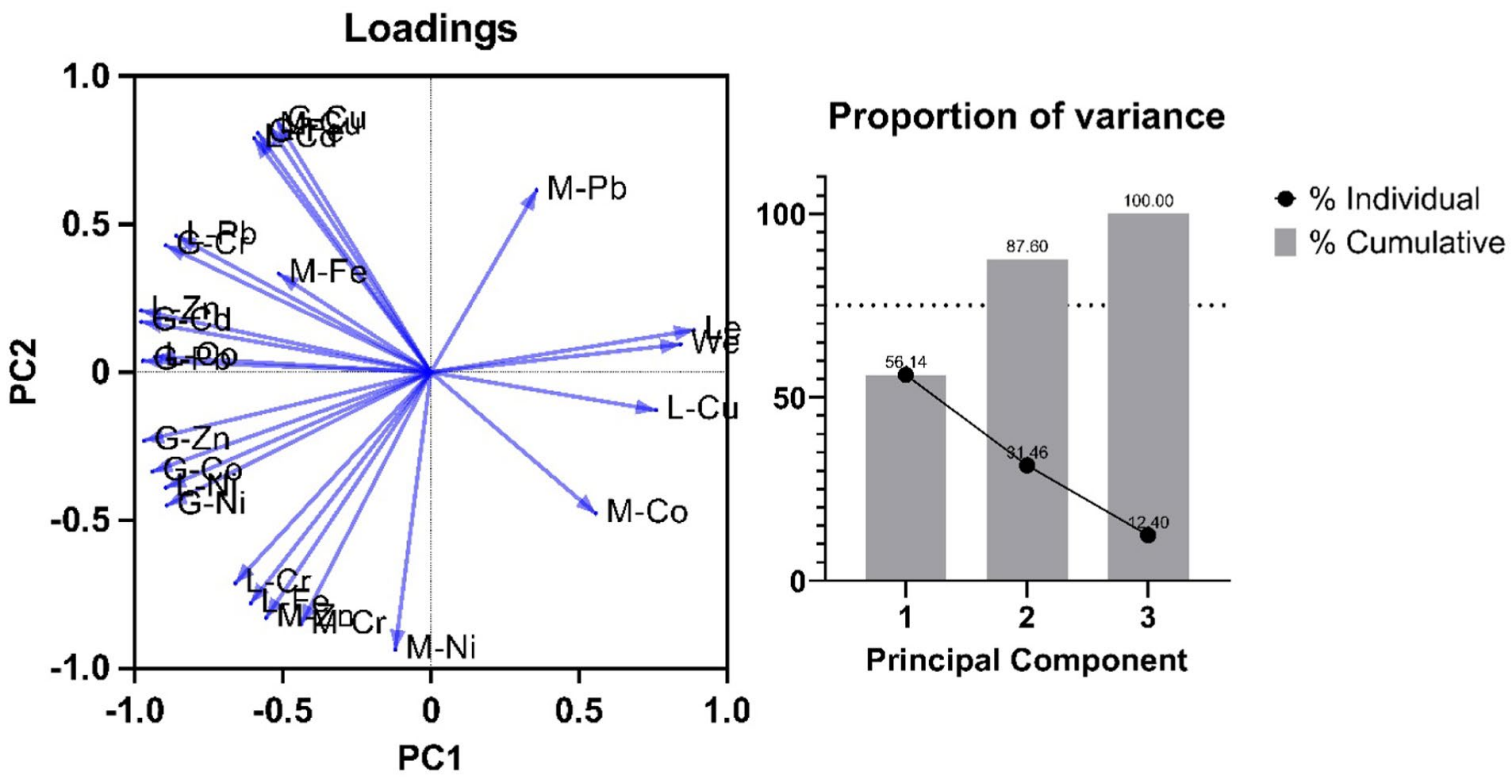
A plot of PCA of heavy metals in various tissue systems (M-Muscle, L-Liver, G-Gills) of *Oreochromis mossambicus*, **(A)** Loadings **(B)** variance covered by individual Principal component.

Two principal components were estimated using the JMP for our dataset comprising of body weight, Length and heavy metal concentration of different organs of fish (Figure 4). Both components together were able to explain ~88% of total variance in the data, with PC1 and PC2 accounting for 56.1 and 31.4%, respectively. The general trend shows negative loading of heavy metals in various fish organs in Tilapia Fish when compared with fish size and body weight. From PC1 we can observe that heavy metal content more specifically in the Gills and Liver is loaded heavily on PC1 and seems to decrease with an increase in the length and weight of the fish. However, the loading of heavy metals in muscles seems to be dependent on both PC1 and PC2, which suggests some other factors influencing the loading apart from the length and weight of the Tilapia fish. This observation corroborates various similar studies performed by researchers over the past decades (52).

### 3.5 Results of correlation matrix analysis (CMA)

**Figure 5:**
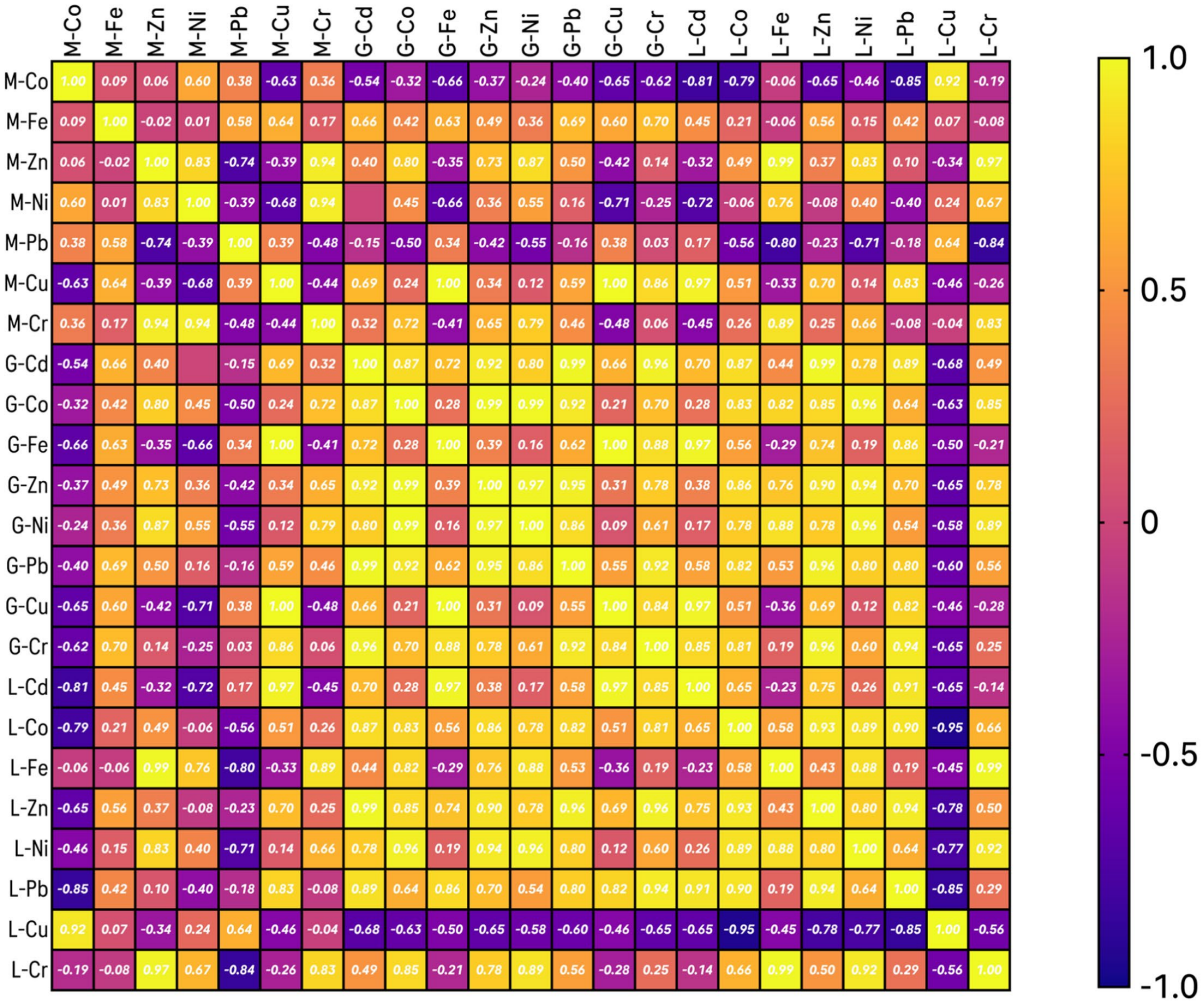
Correlation matrix of the studied metals in various tissue systems of *Oreochromis mossambicus.*

Figure 5 shows the Correlation matrix analysis results of the studied metals in the Tilapia muscle samples: positive and strong significant correlations exist between Cr/Zn and Cr/Ni, also Ni/Zn shows some positive correlation. Considering other organs: in the Liver, positive and strong significant correlations exist between Cd/Pb, Co/Pb, Zn/Pb, Co/Zn, Cr/Fe, and Cr/Ni, also some positive correlation is displayed between Ni/Co. while strong and negative correlation exists between Cu/Co, some negative correlation exists between Pb/Cu, Cu/Zn, and Cu/Ni; in Gills, positive and strong significant correlations were shown by Zn/Cd, Zn/Co, Ni/Co, Ni/Zn, Pb/Cd, Pb/Co, Pb/Zn, Cu/Fe, Cr/Cd, and Cr/Pb, also some positive correlation was shown between Ni/Pb.

## 4 Discussion

Although excessive amounts of iron are linked to heart disease, cancer, and reduced insulin sensitivity, iron is a necessary element for biological activity (53). High iron content in the fish organs could be attributed to the prolonged exposure given that the Cauvery River water iron content is high (54, 55). As per prior studies, Iron content in river water is mostly due to the tributaries from mineralized zones (56). PCA points out that Cu concentration in the Liver of Tilapia loads positively with the length and size of the fish (57). High concentration of Cu in the liver has been demonstrated to have significant poisonous effect on fish (58). The loading of rest of the metals in Liver and Gills shows negative loading when compared to size and weight of the fish. This seems to indicate younger fishes tend to have a higher accumulation of heavy metals than older fishes. This phenomenon has been observed in some earlier studies on other fish species as well. Our study also highlights the higher heavy metal concentration is there in the Gills and Liver of fishes when compared to muscles this is in line with the previous researches in similar area, according to which gills are exposed to the immediate environment and hence more exposed to the heavy metal pollution, on the other hand the Liver is metabolically active and despite the route of exposure be it food or via gills the accumulation of heavy metals take place here (59). Mozambique Tilapia are omnivorous and feed on a variety of food sources, including algae, detritus, and small invertebrates. This diverse diet can lead to the ingestion of metals present in the sediment and water, which can then accumulate in their tissues (60). Muscle however is not metabolically active and thus high heavy metal concentration in Tilapia’s muscles raises concerns. In an earlier study (61), which was performed on three economically important fish species (*Anguilla anguilla, Mugil cephalus, and Oreochromis niloticus*) in Turkey, it was also found that the concentrations of cadmium (Cd) and lead (Pb) were generally lower in the muscles compared to other tissues like the liver and gills, but still present in measurable amounts. The study also highlighted that omnivorous fish like Nile tilapia tend to accumulate metals in their muscles, albeit at different levels depending on the specific metal and environmental conditions.

The strong positive correlation of Chromium (Cr) with Zinc (Zn) and Nickel (Ni) may suggest that these are metals from a common source or with similar pathways of accumulation in the muscle tissue. This can be seen as an indicator of industrial discharge or runoff with these metals. The correlation between Nickel (Ni) and Zinc (Zn) further supports the above hypothesis to have an equal source or has similar environmental behavior, probably from industrial activities or urban runoff.

High correlations between Cadmium (Cd), Lead (Pb), Cobalt (Co), Zinc (Zn), Chromium (Cr), and Iron (Fe) within the liver organ indicate that these metals might be co-contaminants from industrial processes, mining activities, or agricultural runoff (62). It is also likely that the liver organ contains heavy metals because it is a primary detoxifying organ, in which it may accumulate metals reflecting environmental presence. The positive covariance of Nickel (Ni) and Cobalt (Co) could mean that they share a common source, perhaps from either of the metal plating industries or even natural geological sources. The negative correlation of Copper (Cu) and Cobalt (Co) might be interpreted as competition in uptake or different sources. For example, Cu may be more related to agricultural runoff in instances of pesticides (63), whereas Co may be more related to industrial discharge. These negative correlations could be related to other sources or antagonist interactions in the environment itself or within the fish’s biological system.

Strong positive correlations among these metals in gills suggest they should co-exist within the water body, possibly through industrial effluent or urban runoff. As gills are directly exposed to the water, they could indicate water-borne metal contamination. The positive correlation between Nickel and Lead gives further support to a shared source from industrial activity.

The amount of heavy metals like Cr, Co, Pb, and Cd needs to be strictly regulated given these metals have been established as toxic to human health. Studying the Pollution Index shows that the Tilapia fish Muscle and Gills samples are contaminated with Cd, Cr, Fe, Co, Ni, and Pb given the PI > 1. Other examined metals with PI values below 1 include Zn and Cu. This may indicate that the fish samples are free of these metals’ contamination. The correlation matrix indicated a significant relationship between the analyzed metals, suggesting similar sources and/or genetic origin. EDI and THQ for certain metals suggest that prolonged consumption of Tilapia in higher quantities could lead to serious health impacts. Only certain metals such as Cd, Cr, and Pb have been established to have cancer-causing roles in humans. The CR factor for Cd and Cr were found to be higher than the USEPA set tolerable limit. This suggests cancer risk due to Cd and Cr can be there over prolonged exposure. Pb in fish organs was found to have no such risk due to its presence within the tolerable limit. Overall, the data indicates that a higher intake of Tilapia fish might harm the health of the populace consuming it.

## 5 Conclusion and recommendation

Our study looked into a few particular potentially hazardous metals and the risks they pose to human health. According to the metal pollution index values, the amount of contamination in the samples of tilapia fish for Cd, Cr, Fe, Co, Ni, and Pb is greater than 1. The Target hazard quotient for Cd, Co, Pb, and Cr in the Liver, Co, and Cr in Gills, and Co in Muscle were > 1 for adults, which showed a significant health risk other than cancer from the combined effects of these metals. In the muscle, liver, and gills, the cancer risk (Cd and Cr) was higher than the established tolerable level, suggesting that consuming tilapia fish may carry a risk of these heavy metals causing cancer. There is a growing need for more active monitoring regarding the food safety of the Indian population that consumes fish, it would also help generate more data on the state of edible fish species in other Indian rivers. It would be prudent to limit the daily consumption of tilapia to prevent long-term detrimental effects on human health based on the results obtained for the cancer risk. Among the metals taken into consideration, the greatest risk for human health can be associated with the level of Cd and Cr. Further studies and data generation is recommended to study the impact of contaminated fish consumption on the local population over the extended time period.

## 6 Limitations

Although our study offers insightful information about the levels of heavy metals and related hazards in different fish organs, there are a few things to keep in mind. First off, our findings might not be as broadly applicable as they could be because of the sample size and geographic reach, which might not accurately reflect the larger fish population. Furthermore, the results may be impacted by variations in heavy metal buildup brought on by fish species, age, and size that were not fully controlled. Even with its robustness, the risk assessment based on USEPA recommendations might not take into consideration all potential exposure situations and individual susceptibilities, like dietary habits or pre-existing medical disorders. Moreover, possible interactions between various heavy metals that could increase or decrease the total risk were not assessed in the study. Future research should aim to address these limitations by incorporating a larger, more diverse sample set, and by considering additional variables and potential synergistic effects of multiple contaminants.

## Data Availability

The original contributions presented in the study are included in the article/Supplementary material, further inquiries can be directed to the corresponding author/s.
